# International Nosocomial Infection Control Consortium (INICC) national report on device-associated infection rates in 19 cities of Turkey, data summary for 2003–2012

**DOI:** 10.1186/s12941-014-0051-3

**Published:** 2014-11-18

**Authors:** Hakan Leblebicioglu, Nurettin Erben, Victor Daniel Rosenthal, Begüm Atasay, Ayse Erbay, Serhat Unal, Gunes Senol, Ayse Willke, Asu Özgültekin, Nilgün Altin, Mehmet Bakir, Oral Oncul, Gülden Ersöz, Davut Ozdemir, Ata Nevzat Yalcin, Halil Özdemir, Dinçer Yıldızdaş, Iftihar Koksal, Canan Aygun, Fatma Sirmatel, Alper Sener, Nazan Tuna, Özay Arikan Akan, Huseyin Turgut, A Pekcan Demiroz, Tanil Kendirli, Emine Alp, Cengiz Uzun, Sercan Ulusoy, Dilek Arman

**Affiliations:** Ondokuz Mayis University Medical School, Samsun, Turkey; Eskisehir Osmangazi University, Eskisehir, Turkey; International Nosocomial Infection Control Consortium, Ave # 4580, Floor 12, Apt D, Corrientes, Buenos Aires 1195 Argentina; Department of Newborn Medicine, Ankara University School of Medicine, Faculty of Paediatrics, Ankara, Turkey; Turkiye Yuksek Ihtisas Education and Research Hospital, Ankara, Turkey; Hacettepe University School of Medicine, Ankara, Turkey; Suat Seren Chest Diseases and Chest Surgery Training Hospital, Izmir, Turkey; Kocaeli University Faculty of Medicine, Kocaeli, Turkey; Haydarpaşa Numune Training and Research Hospital, Istanbul, Turkey; Etlik İhtisas Training and Education Hospital, Ankara, Turkey; Cumhuriyet University School of Medicine, Sivas, Turkey; Gulhane Military Medical Academy, Haydarpasa Training Hospital, Istanbul, Turkey; Mersin University, Faculty of Medicine, Mersin, Turkey; Duzce University Medical School Infectious Diseases and Clinical Microbiology, Duzce, Turkey; Akdeniz University, Antalya, Turkey; Konya Training and Research Hospital, Konya, Turkey; Çukurova University Balcali Hospital, Adana, Turkey; Karadeniz Technical University School of Medicine, Trabzon, Turkey; Ondokuz Mayis University Medical School (Neonatal Unit), Samsun, Turkey; Abant Izzet Baysal University, Bolu, Turkey; Onsekiz Mart University Canakkale, Canakkale, Turkey; Sakarya Universty, Faculty of Medicine, Sakarya, Turkey; Ankara University School of Medicine Ibni-Sina Hospital, Ankara, Turkey; Pamukkale University, Denizli, Turkey; Ankara Training and Research Hospital, Ankara, Turkey; Department of Paediatric Critical Care Medicine, Ankara University School of Medicine, Ankara, Turkey; Erciyes University, Faculty of Medicine, Kayseri, Turkey; German Hospital, Istanbul, Turkey; Ege University Medical Faculty, Izmir, Turkey; Gazi University Medical School, Ankara, Turkey

**Keywords:** Hospital infection, Nosocomial infection, Healthcare-associated infection, INICC, International Nosocomial Infection Consortium, Turkey, Device-associated infection, Antibiotic resistance, Ventilator-associated pneumonia, Catheter-associated urinary tract infection, Central line-associated bloodstream infections, Bloodstream infection, Urinary tract infection, Network

## Abstract

**Background:**

Device-associated healthcare-acquired infections (DA-HAI) pose a threat to patient safety, particularly in the intensive care unit (ICU). We report the results of the International Infection Control Consortium (INICC) study conducted in Turkey from August 2003 through October 2012.

**Methods:**

A DA-HAI surveillance study in 63 adult, paediatric ICUs and neonatal ICUs (NICUs) from 29 hospitals, in 19 cities using the methods and definitions of the U.S. NHSN and INICC methods.

**Results:**

We collected prospective data from 94,498 ICU patients for 647,316 bed days. Pooled DA-HAI rates for adult and paediatric ICUs were 11.1 central line-associated bloodstream infections (CLABSIs) per 1000 central line (CL)-days, 21.4 ventilator-associated pneumonias (VAPs) per 1000 mechanical ventilator (MV)-days and 7.5 catheter-associated urinary tract infections (CAUTIs) per 1000 urinary catheter-days. Pooled DA-HAI rates for NICUs were 30 CLABSIs per 1000 CL-days, and 15.8 VAPs per 1000 MV-days. Extra length of stay (LOS) in adult and paediatric ICUs was 19.4 for CLABSI, 8.7 for VAP and 10.1 for CAUTI. Extra LOS in NICUs was 13.1 for patients with CLABSI and 16.2 for patients with VAP. Extra crude mortality was 12% for CLABSI, 19.4% for VAP and 10.5% for CAUTI in ICUs, and 15.4% for CLABSI and 10.5% for VAP in NICUs. Pooled device use (DU) ratios for adult and paediatric ICUs were 0.54 for MV, 0.65 for CL and 0.88 for UC, and 0.12 for MV, and 0.09 for CL in NICUs. The CLABSI rate was 8.5 per 1,000 CL days in the Medical Surgical ICUs included in this study, which is higher than the INICC report rate of 4.9, and more than eight times higher than the NHSN rate of 0.9. Similarly, the VAP and CAUTI rates were higher compared with U.S. NHSN (22.3 vs. 1.1 for VAP; 7.9 vs. 1.2 for CAUTI) and with the INICC report (22.3 vs. 16.5 in VAP; 7.9 vs. 5.3 in CAUTI).

**Conclusions:**

DA-HAI rates and DU ratios in our ICUs were higher than those reported in the INICC global report and in the US NHSN report.

## Background

Increasingly in scientific literature, DA-HAIs are considered to be among the principal threat to patient safety in the ICU and are among the main causes of patient morbidity and mortality [1,2].

The effectiveness of implementing an integrated infection control programme focused on device-associated healthcare-acquired infection (DA-HAI) surveillance was demonstrated in the many studies conducted in the U.S., whose results reported not only that the incidence of DA-HAI can be reduced by as much as 30%, but that a related reduction in healthcare costs was also feasible [3]. In the same way, it is fundamental to address the burden of antimicrobial-resistant infections that the pathogens and the susceptibility to antimicrobials of DA-HAI-associated pathogens be reported, so that informed decisions can be made to effectively prevent transmission of resistant strains and their determinants, such as strains with phenotypes with very few available treatments with chances of success [4].

For more than 30 years, the U.S. the Centers for Disease Control and Prevention (CDC)’s National Healthcare Safety Network (NHSN) [5] has provided benchmarking U.S. ICU data on DA-HAIs, which have proven invaluable for researchers [5], and served as an inspiration to the INICC [6]. The INICC is an international non-profit, open, multi-centre, collaborative healthcare-associated infection control programme with a surveillance system based on that of the CDC’s NHSN [5]. Founded in Argentina in 1998, INICC is the first multinational research network established to measure, control and reduce DA-HAI in ICUs and surgical site infections (SSIs) hospital wide through the analysis of data collected on a voluntary basis by a pool of hospitals worldwide [6,7]. The INICC has the following goals: To create a dynamic global network of hospitals worldwide and conduct surveillance of DA-HAIs and SSIs using standardized definitions and established methodologies, to promote the implementation of evidence-based infection control practices, and to carry out applied infection control research; to provide training and surveillance tools to individual hospitals which can allow them to conduct outcome and process surveillance of DA-HAIs and SSIs, to measure their consequences, and assess the impact of infection control practices; to improve the safety and quality of healthcare world-wide through the implementation of systematized programmes to reduce rates of DA-HAIs and SSIs, their associated mortality, excess lengths of stay (LOS), excess costs, antibiotic usage, and bacterial resistance [8].

This report is a summary of data on DA-HAIs collected in 63 intensive care units (ICUs) in 29 Turkish hospitals from 19 cities participating in the International Nosocomial Infection Control Consortium (INICC) between August 2003 and October 2012 [6,7].

## Methods

### Setting and study design

This prospective cohort surveillance study was conducted in 63 adult, paediatric ICUs and neonatal ICUs (NICUs) from 29 hospitals in 19 cities. Hospitals were stratified by bed numbers (<200, 201–500, 501–1000, and >1000).

The ICUs were stratified according to the patient features: adult, paediatric or NICUs. The types of ICU participating in this study were the following: Cardiothoracic, Medical, Medical Cardiac, Medical/Surgical, Neurologic, Neurosurgical, Neonatal, Paediatric, Respiratory and Surgical.

According to the level of complexity of care, the NICUs included the following levels:

- Level IIIA: It provides care to neonatal patients born at ≥28 weeks, who weigh ≥1,000 grams. The provide mechanical ventilation and minor surgical procedures, such as umbilical vessel catheterization.
- Level IIIB: It provides care to neonatal patients born at any viable gestational age. Mechanical ventilation and high-frequency mechanical ventilation are provided. There are paediatric surgical centres on site or nearby to complete major surgical procedures.
- Level IIIC: It provides the highest level of NICU care. In addition to the capabilities of Level IIIA and B, it provides extra corporeal membrane oxygenation and complicated surgical procedures requiring cardiopulmonary bypass are performed as well.

### INICC methodology

The INICC is focused on the surveillance and prevention of DA-HAI in adult, paediatric ICUs and neonatal ICUs (NICUs), and of SSIs in surgical procedures hospital wide [6,7]. The INICC has both outcome surveillance and process surveillance components. The modules of the components may be used singly or simultaneously, but, once selected; they must be used for a minimum of 1 calendar month. All DA-HAIs and SSIs of the Outcome Surveillance Component are categorized using standard NHSN definitions that include laboratory tests, radiology tests, and clinical criteria [9]. Laboratory-confirmed BSIs are recorded and reported [9].

The Outcome Surveillance Component related to DA-HAI classifies surveillance data into specific module protocols that include excess LOS, evaluation of DA-HAI costs, crude excess length of stay, crude excess mortality, microbiological profile, bacterial resistance, and antimicrobial-use data. Data on DA-HAI costs were not included in this report. Data from the INICC Process Surveillance Module, which includes monitoring of hand hygiene, vascular catheter care, urinary catheter care, and mechanical ventilator care compliance, were not included in this report.

### Training, validation, and reporting

The INICC Chairman trained the principal and secondary investigators at hospitals. Investigators were also provided with a manual and training tool that described in detail how to perform surveillance and complete surveillance forms. In addition, investigators had continuous e-mail and telephone access to a support team at the INICC Central Office in Buenos Aires, Argentina.

Each month, participating hospitals submitted the completed surveillance forms to the INICC Central Office, where the validity of each case was checked and the recorded signs and symptoms of infection and the results of laboratory studies, radiographic studies, and cultures were scrutinized to assure that the U.S. NHSN criteria for DA-HAI had been met. The forms used for surveillance of each ICU patient permit both internal and external validation, because they include every clinical and microbiological criterion for each type of DA-HAI [6,8]. Therefore, the investigator who reviewed the data forms filled in at the participating hospital verified that adequate criteria for infection had been fulfilled in each case; and the original patient data form was further validated at the INICC Central Office before data on the reported infection are entered into the INICC’s database.

### Data collection

Using standardized INICC detailed forms and following the INICC protocol and U.S. NHSN’s definitions [9], infection control professionals (ICPs), trained and with previous experience conducting surveillance of DA-HAIs, collected data on central line-associated bloodstream infections (CLABSIs), catheter-associated urinary tract infections (CAUTIs) and ventilator-associated pneumonias (VAPs) in the ICUs.

In the NICUs, ICPs collected data on CLABSIs and umbilical catheter-associated primary bloodstream infections or VAPs for each of 5 birth-weight categories (<750 g, 750–1000 g, 1001 – 1500 g, 1501 – 2500 g, >2500 g), Corresponding denominator data, patient-days and specific device-days were also collected by the ICPs.

Detailed and aggregated data were used to calculate DA-HAI rates per 1000 device-days. Only prospective data using INICC patient detailed forms were used to calculate mortality and LOS.

In accordance with the INICC’s Charter, the identity of all INICC hospitals and cities is kept confidential.

### Data analysis

Data for adult combined medical/surgical ICUs were not stratified by type or size of hospital. Data for NICUs were stratified by weight categories: central line-days, urinary catheter-days, or ventilator days.

Device-days consisted of the total number of central line (CL)-days, urinary catheter (UC)-days, or mechanical ventilator (MV)-days. For NICUs, device-days consisted of the total number of CL-days, UC-days, and MV-days.

Crude excess mortality of DA-HAI equals crude mortality of ICU patients with DA-HAI minus crude mortality of patients without DA-HAI.

Crude excess LOS of DA-HAI equals crude LOS of ICU patients with DA-HAI minus crude LOS of patients without DA-HAI.

Comparisons of the percentile distribution were made if there were at least 7 locations contributing to the strata.

EpiInfo® version 6.04b (CDC, Atlanta, GA) and SPSS 16.0 (SPSS Inc. an IBM company, Chicago, Illinois) were used to conduct data analysis. Relative risk (RR) ratios, 95% confidence intervals (CIs) and P-values were determined for primary and secondary outcomes.

## Results

The characteristics of 63 ICUs from 29 hospitals in 19 cities from Turkey currently participating in INICC that contributed data for this report are shown in Table 1. The length of hospital’s participation in the INICC Programme is as follows: mean length of participation ± SD, 28.7 ± 25.7 months, range 3 to 85 months.

**Table 1:** **Characteristics of the participating intensive care units**

	**<200 beds hospitals**	**201-500 bed hospitals**	**501-1000 bed hospitals**	**>1000 bed hospitals**	**Overall**
No. of hospitals	3 (10%)	8 (28%)	10 (34%)	8 (28%)	29 (100%)
No. of ICUs	4 (6%)	20 (32%)	29 (46%)	10 (16%)	63 (100%)
Medical Cardiac	1 (25%)	2 (50%)	1 (25%)	0 (0%)	4 (100%)
Cardiothoracic	0 (0%)	1 (33%)	1 (33%)	1 (33%)	3 (100%)
Medical	0 (0%)	4 (44%)	3 (33%)	2 (22%)	9 (100%)
Medical/Surgical	1 (5%)	5 (26%)	9 (47%)	4 (21%)	19 (100%)
Neonatal	1 (17%)	2 (33%)	2 (33%)	1 (17%)	6 (100%)
Neurologic	0 (0%)	0 (0%)	2 (100%)	0 (0%)	2 (100%)
Neurosurgical	0 (0%)	1 (33%)	2 (67%)	0 (0%)	3 (100%)
Paediatric	1 (14%)	1 (14%)	4 (57%)	1 (14%)	7 (100%)
Respiratory	0 (0%)	1 (50%)	1 (50%)	0 (0%)	2 (100%)
Surgical	0 (0%)	3 (38%)	4 (50%)	1 (13%)	8 (100%)

ICU, intensive care unit.

For the Outcome Surveillance Component, DA-HAI rates, device utilization (DU) ratios, crude excess mortality by specific type of DA-HAI, microorganism profile and bacterial resistance from August 2003 through October 2012 are summarized (Tables 2, 3, 4, 5, 6, 7, 8, 9, 10, 11, 12 and 13).

**Table 2:** **Pooled means of central line-associated bloodstream infection rates, urinary catheter-associated urinary tract infection rates, and ventilator-associated pneumonia by hospital size**

**Hospital size, beds, n**	**ICUs, n**	**Patients, n**	**Bed days, n**	**CL days, n**	**CLABSI, n**	**CLABSI rate (95% CI)**	**MV days, n**	**VAP, n**	**VAP, Rate (95% CI)**	**UC days, n**	**CAUTI, n**	**CAUTI, rate (95% CI)**
<200	3	713	14 706	9,459	41	4.3 (31 – 5.9)	7,536	40	5.3 (3.8 - 7.2)	10 621	43	4.0 (2.9 - 5.5)
201-500	18	23 896	167 058	88 917	382	4.3 (3.9 – 4.7)	84 714	2193	25.9 (24.8 - 26.9)	142 965	652	4.6 (4.2 - 4.9)
501-1000	27	61 350	382 283	189 728	1,939	10.2 (9.8 – 10.7)	142 735	3152	22.1 (21.3 - 22.8)	314 847	2957	9.4 (9.0 - 9.7)
>1000	9	5,109	4,914	31 432	329	10.5 (9.4 – 11.7)	37 310	431	11.6 (10.4 - 12.7)	42 106	180	4.3 (3.7 - 4.9)
**Pooled**	57	91 068	613,191	319 536	2,691	8.4 (8.1 – 8.7)	272 295	5,816	21.4 (20.8 - 21.9)	510 539	3,832	7.5 (7.3 - 7.7)

Adult and Paediatric Patients. DA module, 2003-2012

ICU, intensive care units; CL, central line; CLABSI, central line-associated bloodstream infection; CI, confidence interval; MV, mechanical ventilator; VAP, ventilator-associated pneumonia; UC, urinary catheter; CAUTI, catheter-associated urinary tract infection.

**Table 3:** **Pooled means of central line-associated bloodstream infection rates, and ventilator-associated pneumonia by hospital size**

**Hospital size, beds, n**	**ICUs, n**	**Patients, n**	**Bed days, n**	**CL days**	**CLABSI, N**	**CLABSI rate (95% CI)**	**MV days, n**	**VAP, n**	**VAP, rate (95% CI)**
<200	1	440	4,457	269	29	107.8 (72.2 – 154.8)	273	11	40.3 (20.2 - 70.9)
201-500	2	383	4,834	1706	6	3.5 (1.3 – 7.7)	1,206	19	15.8 (9.0 - 24.5)
501-1000	2	1,442	16 826	2206	51	23.1 (17.2 – 30.4)	3,046	28	9.2 (6.1 - 13.2)
>1000	1	1,165	8,008	1049	24	22.9 (14.7 – 34.0)	985	29	29.4 (19.8 - 42.0)
**Pooled**	6	3,430	34 125	5,230	110	21.0 (17.3 – 25.3)	5,510	87	15.8 (12.6 - 19.5)

Neonatal Patients. DA module, 2003–2012.

ICU, intensive care units; CL, central line; CLABSI, central line-associated bloodstream infection; CI, confidence interval; MV, mechanical ventilator; VAP, ventilator-associated pneumonia.

**Table 4:** **Pooled means and key percentiles of the distribution of central line-associated bloodstream infection rates, by type of location, adult and paediatric patients**

**Type of ICU**	**ICU, n**	**Patients**	**Bed days**	**CL days**	**CLABSI, n**	**CLABSI rate**	**95% CI**	Percentiles*				
								**10**	**25**	**50**	**75**	**90**
Medical Cardiac	4	5,380	22 743	10 838	46	4.2	3.1 – 5.7	-	-	-	-	-
Cardiothoracic	3	7,800	21 796	15 165	22	1.5	0.9 – 2.2	-	-	-	-	-
Medical	9	21 854	170 042	79 343	525	6.6	6.1 – 7.2	2.5	3.8	7.3	11.1	-
Medical/Surgical	19	19 410	175 470	113 597	969	8.5	8.0 – 9.1	0.0	4.2	11.7	15.1	18.3
Neurologic	2	3,784	30 966	8,690	91	10.5	8.4 – 12.9	-	-	-	-	-
Neurosurgical	3	5,691	39 719	18 579	103	5.5	4.5 – 6.7	-	-	-	-	-
Paediatric	7	4,235	32 148	12 880	122	9.5	7.9 – 11.3	0.0	2.7	10.6	13.6	-
Respiratory	2	1,754	14 054	4,950	59	11.9	9.1 – 15.4	-	-	-	-	-
Surgical	8	21 160	106 253	55 494	754	13.6	12.6 – 14.6	1.6	3.5	9.8	17.2	-
**Pooled**	57	91 068	613 191	319 536	2,691	8.4	8.1 – 8.7	1.0	3.9	8.6	13.8	18.2

DA module, 2003–2012.

ICU, intensive care unit; CL, central line; CLABSI, central line-associated bloodstream infection; CI, confidence interval.

*Comparisons of the percentile distribution were made if there were at least 7 locations contributing to the strata.

**Table 5:** **Pooled means of the distribution of central line-associated bloodstream infection rates for level III NICUs, stratified by birth-weight category**

**Birth-weight category**	**ICU, n**	**Patients**	**Bed days**	**CL days**	**CLABSI, n**	**CLABSI rate**	**95% CI**
<750 grams	4	98	617	250	9	36.0	16.5 – 68.3
751-1000 grams	6	297	4,197	1,639	30	18.3	12.3 – 26.1
1001-1500 grams	6	649	10 652	1,465	48	32.8	24.2 – 43.4
1501-2500 grams	6	1,202	10 998	1,024	8	7.8	3.4 – 15.4
>2500 grams	6	1,184	7,661	852	15	17.6	9.9 – 29.0
**Pooled**	6	3,430	34 125	5,230	110	21.0	17.3 – 25.3

DA module, 2003–2012.

ICU, intensive care unit; CL, central line; CLABSI, central line-associated bloodstream infection; CI, confidence interval.

**Table 6:** **Pooled means and key percentiles of the distribution of ventilator-associated pneumonia rates, by type of location, adult and paediatric patients**

**Type of ICU**	**ICUs, n**	**Patients**	**Bed days**	**MV days**	**VAP, n**	**VAP rate**	**95% CI**	Percentiles*				
								**10**	**25**	**50**	**75**	**90**
Medical Cardiac	4	5, 380	22 743	5,820	58	10.0	7.6 –12.9	-	-	-	-	-
Cardiothoracic	3	7,800	21 796	9,993	123	12.3	10.2 – 14.7	-	-	-	-	-
Medical	9	21 854	170 042	82 378	1836	22.3	21.3 – 23.3	8.3	12.6	22.1	32.7	-
Medical/Surgical	19	19 410	175 470	95 021	2116	22.3	21.3 – 23.2	9.6	12.8	16.5	28.6	42.9
Neurologic	2	3,784	30 966	7,405	176	23.8	20.4 – 27.6	-	-	-	-	-
Neurosurgical	3	5,691	39 719	8,859	252	28.4	25.0 – 32.2	-	-	-	-	-
Paediatric	7	4,235	32 148	17 068	200	11.7	10.2 – 13.5	2.9	6.2	10.6	14.1	-
Respiratory	2	1,754	14 054	8,156	204	25.0	21.7 – 28.7	-	-	-	-	-
Surgical	8	21 160	106 253	37 595	851	22.6	21.1 – 24.2	12.6	18.5	21.9	26.7	-
**Pooled**	57	91 068	613 191	272 295	5,816	21.4	20.8 – 21.9	7.2	11.2	20.5	27.7	35.4

DA module, 2003–2012.

ICU, intensive care unit; MV, mechanical ventilator; VAP, ventilator-associated pneumonia; CI, confidence interval.

*Comparisons of the percentile distribution were made if there were at least 7 locations contributing to the strata.

**Table 7:** **Pooled means of the distribution of ventilator-associated pneumonia rates for level III NICUs, stratified by Birth-weight category**

**Birth-weight category**	**ICUs, n**	**Patients**	**Bed days**	**MV days**	**VAP, n**	**VAP rate**	**95% CI**
<750 grams	4	98	617	236	4	16.9	4.6 – 43.4
751-1000 grams	6	297	4197	1,407	25	17.8	11.5 – 26.2
1001-1500 grams	6	649	10 652	1,307	19	14.5	8.8 – 22.7
1501-2500 grams	6	1,202	10 998	1,318	19	14.4	8.7 – 22.5
>2500 grams	6	1,184	7,661	1,242	20	16.1	9.8 – 24.9
**Pooled**	6	3,430	34 125	5,510	87	15.8	12.6 – 19.5

DA module, 2003-2012.

ICU, intensive care unit; MV, mechanical ventilator; VAP, ventilator-associated pneumonia; CI, confidence interval.

**Table 8:** **Pooled means and key percentiles of the distribution of urinary catheter-associated urinary tract infection rates, by type of location, adult and paediatric patients**

**Type of ICU**	**ICU, n**	**Patients**	**Bed days**	**UC days**	**CAUTI, n**	**CAUTI, rate**	**95% CI**	Percentiles*				
								**10**	**25**	**50**	**75**	**90**
Medical Cardiac	4	5,380	22 743	14 907	49	3.3	2.4 - 4.3	-	-	-	-	-
Cardiothoracic	3	7,800	21 796	18 744	68	3.6	2.8 - 4.6	-	-	-	-	-
Medical	9	21 854	170 042	143 455	739	5.2	4.8 - 5.5	2.1	2.8	4.0	8.9	-
Medical/Surgical	19	19 410	175 470	154 422	1,220	7.9	7.5 - 8.4	2.1	2.8	5.8	9.1	13.7
Neurologic	2	3,784	30 966	29 856	596	20.0	18.4 - 21.6	-	-	-	-	-
Neurosurgical	3	5,691	39 719	36 688	347	9.5	8.5 - 10.5	-	-	-	-	-
Paediatric	7	4,235	32 148	10 981	73	6.6	5.2 - 8.4	1.1	1.8	3.9	10.7	-
Respiratory	2	1,754	14 054	12 833	50	3.9	2.9 - 5.1	-	-	-	-	-
Surgical	8	21 160	106 253	88 653	690	7.8	7.2 - 8.4	1.7	2.8	5.5	8.9	-
**Pooled**	57	91 068	613 191	510 539	3,832	7.5	7.3 - 7.7	1.7	2.6	4.9	8.5	14.2

DA module, 2003–2012.

ICU, intensive care unit; UC, urinary catheter; CAUTI, catheter-associated urinary tract infection; CI, confidence interval.

*Comparisons of the percentile distribution were made if there were at least 7 locations contributing to the strata.

**Table 9:** **Pooled means of the distribution of central line utilization ratios, urinary catheter utilization ratios, and ventilator utilization ratios, by type of location, adult and paediatric patients**

**ICU type**	**ICU, n**	**Bed days**	**CL days**	**DUR, central line (95% CI)**	**MV days**	**DUR, MV (95% CI)**	**UC days**	**DUR, UC (95% CI)**
Medical Cardiac	4	22 743	10 838	0.48 (0.47 – 0.48)	5,820	0.26 (0.25 – 0.26)	14 907	0.66 (0.65 – 0.66)
Cardiothoracic	3	21 796	15 165	0.70 (0.69 – 0.70)	9,993	0.46 (0.45 – 0.47)	18 744	0.86 (0.86 – 0.86)
Medical	9	170 042	79 343	0.47 (0.46 – 0.47)	82 378	0.48 (0.48 – 0.49)	143 455	0.84 (0.84 – 0.85)
Medical/Surgical	19	175 470	113 597	0.65 (0.65 – 0.65)	95 021	0.54 (0.54 – 0.54)	154 422	0.88 (0.88 – 0.88)
Neurologic	2	30 966	8,690	0.28 (0.28 – 0.29)	7,405	0.24 (0.23 – 0.24)	29 856	0.96 (0.96 – 0.97)
Neurosurgical	3	39 719	18 579	0.47 (0.46 – 0.47)	8,859	0.22 (0.22 – 0.23)	36 688	0.92 (0.92 – 0.93)
Paediatric	7	32 148	12 880	0.40 (0.40 – 0.41)	17 068	0.53 (0.53 – 0.54)	10 981	0.34 (0.34 – 0.35)
Respiratory	2	14 054	4,950	0.35 (0.34 – 0.36)	8,156	0.58 (0.57 – 0.59)	12 833	0.91 (0.91 – 0.92)
Surgical	8	106 253	55 494	0.52 (0.52 – 0.53)	37 595	0.35 (0.35 – 0.36)	88 653	0.83 (0.83 – 0.84)
**Pooled**	57	613 191	319 536	0.52 (0.52 – 0.52)	272 295	0.44 (0.44 – 0.45)	510 539	0.83 (0.83 – 0.83)

DA module, 2003–2012.

ICU, intensive care unit; CL, central line; MV, mechanical ventilator; UC, urinary catheter; DUR, device use ratio; CI, confidence interval.

**Table 10:** **Pooled means of the distribution of central line utilization ratios and ventilator utilization ratios, by type of location, for level III NICUs**

**Birth-weight category**	**ICU, n**	**Bed days**	**CL days**	**DUR, central line (95% CI)**	**MV days**	**DUR, MV (95% CI)**
<750 grams	4	617	250	0.41 (0.37 – 0.45)	236	0.38 (0.34 – 0.42)
751-1000 grams	6	4197	1639	0.39 (0.38 – 0.41)	1407	0.34 (0.32 – 0.35)
1001-1500 grams	6	10652	1465	0.14 (0.13 – 0.14)	1307	0.12 (0.12 – 0.13)
1501-2500 grams	6	10998	1024	0.09 (0.09 – 0.10)	1318	0.12 (0.11 – 0.13)
>2500 grams	6	7661	852	0.11 (0.10 – 0.12)	1242	0.16 (0.15 – 0.17)
<750 grams	6	34125	5230	0.15 (0.15 – 0.16)	5510	0.16 (0.16 – 0.17)

DA module, 2003–2012.

ICU, intensive care unit; CL, central line, MV, mechanical ventilator; DUR, device use ratio; CI, confidence interval.

**Table 11:** **Pooled means of the distribution of crude mortality and crude excess mortality of adult and paediatric intensive care unit patients with and without device-associated healthcare-acquired infection**

**Adult and paediatric ICUs combined**	**No. of deaths**	**No. of patients**	**Pooled crude mortality, % (95% CI)**	**RR (95% CI)**
Crude mortality of patients without DA-HAI	1,616	6,408	25.2 (24.1- 26.3)	1.0
Crude mortality of patients with CLABSI	133	357	37.3 (32.2- 42.4)	1.5 (1.2 – 1.8)
Crude excess mortality of patients with CLABSI	133	357	12.0 (8.1- 16.1)	-
Crude mortality of patients with CAUTI	55	154	35.7 (28.1- 43.8)	1.4 (1.1 – 1.9)
Crude excess mortality of patients with CAUTI	55	154	10.5 (4.0- 17.5)	-
Crude mortality of patients with VAP	253	567	44.6 (40.4- 48.8)	1.8 (1.6 – 2.0)
Crude excess mortality of patients with VAP	253	567	19.4 (16.3- 22.5)	-
**Neonatal ICUs combined**	**No. of deaths**	**No. of patients**	**Pooled crude mortality, % (95% CI)**	
Crude mortality of patients without DA-HAI	68	1,964	3.5 (2.7- 4.4)	1.0
Crude mortality of patients with CLABSI	10	53	18.9 (9.4- 32.7)	5.5 (2.8 – 10.6)
Crude excess mortality of patients with CLABSI	10	53	15.4 (6.7- 28.3)	-
Crude mortality of patients with VAP	6	43	14.0 (5.3- 27.9)	4.0 (1.8 – 9.3)
Crude excess mortality of patients with VAP	6	43	10.5 (2.6- 23.5)	-

ICU, intensive care units; CI, confidence interval; DA-HAI, device-associated healthcare-acquired infection; CLABSI, central line-associated bloodstream infection; VAP, ventilator-associated pneumonia; CAUTI, catheter-associated urinary tract infection; RR, relative risk.

**Table 12:** **Pooled means of the distribution of the length of stay and crude excess length of stay of intensive care unit patients with and without device-associated healthcare-acquired infection**

**Adult and paediatric ICUs combined**	**LOS, total days**	**No. of patients**	**Pooled average. LOS, days (95% CI)**	**RR (95% CI)**
LOS of patients without DA-HAI	50 716	6,408	7.9 (7.8-7.9)	
LOS of patients with CLABSI	6,920	357	19.4 (17.5-21.6)	2.4 (2.4 – 2.5)
Extra LOS of patients with CLABSI	6,920	357	11.5 (9.7-13.7)	
LOS of patients with CAUTI	2,769	154	18.0 (15.4-21.2)	2.3 (2.2 – 2.3)
Extra LOS of patients with CAUTI	2,769	154	10.1 (7.6-13.3)	
LOS of patients with VAP	9,426	567	16.6 (15.3-18.1)	2.1 (2.0 – 2.1)
Extra LOS of patients with VAP	9,426	567	8.7 (7.5-10.2)	
**Neonatal ICUs combined**	**LOS, total days**	**No. of patients**	**Pooled average LOS, days**	
LOS of patients without DA-HAI	17,547	1,964	8.9 (8.5-9.3)	
LOS of patients with CLABSI	1,169	53	22.1 (16.9-29.5)	2.6 (2.3 – 2.6)
Extra LOS of patients with CLABSI	1,169	53	13.1 (16.9-9.5)	
LOS of patients with VAP	1,081	43	25.1 (18.7-35.7)	2.8 (2.6 – 3.0)
Extra LOS of patients with VAP	1,081	43	16.2 (18.7-35.7)	

LOS, length of stay; DA-HAI, device-associated healthcare-acquired infection; CLABSI, central line-associated bloodstream infection; VAP, ventilator-associated pneumonia; CAUTI, catheter-associated urinary tract infection.

**Table 13:** **Antimicrobial resistance rates in the participating intensive care units**

	**Pathogenic isolated tested, pooled, n**	**Resistance, %**	**Pathogenic isolated tested, pooled, n**	**Resistance, %**	**Pathogenic isolated tested, pooled, n**	**Resistance, %**
**Pathogen, antimicrobial**	**(CLABSI)**	**(CLABSI)**	**(VAP)**	**(VAP)**	**(CAUTI)**	**(CAUTI)**
*Staphylococcus aureus*						
Oxacilin	478	92.7%	482	83.2%	22	81.8%
*Coagulase- negative staphylococci*						
Oxacilin	516	90.3%	69	81.2%	14	71.4%
*Enterococcus faecalis*						
Vancomycin	80	5.0%	10	0.0%	36	0.0%
*Pseudomonas aeruginosa*						
Ciprofloxacine	201	35.3%	719	40.6%	89	36.0%
Piperacillin or piperacillin-tazobactam	279	27.6%	1,009	33.8%	124	31.5%
Amikacin	185	18.9%	671	18.3%	81	16.0%
Imipenem or meropenem	251	37.1%	989	41.0%	122	33.6%
*Klebsiella pneumoniae*						
Ceftriaxone or ceftazidime	140	55.7%	160	46.3%	28	50.0%
Imipenem or meropenem	189	6.3%	224	4.5%	73	1.4%
*Acinetobacter baumanii*						
Imipenem or meropenem	469	56.1%	844	62.8%	73	57.5%
*Escherichia Coli*						
Ceftriaxone or ceftazidime	67	55.2%	77	44.2%	78	51.3%
Imipenem or meropenem	68	4.4%	141	3.5%	132	2.3%
Ciprofloxacine	65	66.2%	110	50.0%	104	33.7%

CLABSI, central line-associated bloodstream infection; VAP, ventilator-associated pneumonia; CAUTI, catheter-associated urinary tract infection.

Table 2 shows DA-HAI rates by infection type (CLABSI, CAUTI, VAP) in adult and paediatric ICUs stratified by hospital size and Table 3 shows the same information regarding NICUs. In adult and paediatric patients, we found higher rates of CLABSI in the largest hospitals (>500 beds), however, VAP and CAUTI rates were higher in middle-sized hospitals (201–1000 beds). In NICU patients the rates of CLABSI and VAP were higher in the smallest hospitals (<200 beds).

Tables 4, 5, 6, 7 and 8 show DA-HAI rates in all the participating ICUs, and in those cases that include NICU patients (Tables 5 and 7), the information is divided by weight category. We found that in adult and paediatric patients the highest CLABSI rate was found in the Surgical ICUs, the highest VAP rate in Neurosurgical ICU, and the highest CAUTI rate in Neurologic ICUs. Regarding NICU patients, the highest CLABSI rate was found in patients within the 1000–1500 grams weight category, and the highest VAP rate was found in patients in the 751–1000 grams weight category.

Tables 9 and 10 provide data on device use ratios (DURs) for CL, UC and MV and their respective confidence intervals. Central line DUR was higher in the cardiothoracic ICUs, the mechanical ventilator DUR was higher in respiratory ICUs, and the urinary catheter DUR was higher in neurologic ICUs. In the NICU patients the highest DUR for central line and mechanical ventilator were found in <750 grams birth weight category.

Table 11 provides data on crude ICU mortality in patients hospitalized in each type of unit during the surveillance period, with and without DA-HAI, and crude excess mortality of adult and paediatric patients with CLABSI, CAUTI, and VAP, and infants in NICUs with CLABSI or VAP. The DA-HAI associated with a higher mortality was VAP in adult and paediatric patients and CLABSI in NICU patients.

Table 12 provides data on crude LOS of patients hospitalized in each ICU during the surveillance period with and without DA-HAI and crude excess LOS of adult and paediatric patients with CLABSI, CAUTI, and VAP and infants in NICUs with CLABSI or VAP. The DA-HAI associated with a longer LOS was CLABSI in adult and paediatric patients and VAP in NICU patients.

Table 13 provides data on bacterial resistance of pathogens isolated from patients with DA-HAI in adult and paediatric ICUs and NICUs. We found a high resistance of *Staphylococci aureus* and Coagulase-negative staphylococci to oxacilin in CLABSIs, VAP and CAUTIs.

Tables 14 and 15 compare the results of this report from Turkey with the INICC international report for the period 2007–2012 and with NHSN report of 2011 [5,10]. Overall, we found higher DA-HAI rates in this study than in INICC and NHSN data, as shown in Table 14. DUR was higher in most cases as well, but the central line DUR was lower in paediatric ICUs and NICUs compared to NHSN. Table 15 compares the antimicrobial resistance rates of this report from Turkey with the INICC international report for the period 2007–2012 and with NHSN report of 2010–2012. In most cases, we found higher resistance rates than those found in the NHSN report.

**Table 14:** **Benchmarking of device-associated healthcare-acquired infection rates in this report against the report of the International Nosocomial Infection Control Consortium (2007–20012) and the report of the US National Healthcare Safety Network Data (2011)**

	**This report**	**INICC report (2007–2012) [** 10 **]**	**U.S. NHSN report (2011) [** 5 **]**
**Medical surgical ICU**			
CL, DUR	0.65 (0.65 – 0.65)	0.54 (0.54 – 0.54)	0.35 (0.35 – 0.35)
CLABSI rate	8.5 (8.0 – 9.1)	4.9 (4.8 – 5.1)	0.9 (0.8 - 0.9)
MV, DUR	0.54 (0.54 – 0.54)	0.36 (0.36 – 0.36)	0.24 (0.24 – 0.24)
VAP rate	22.3 (21.3 - 23.2)	16.5 (16.1 – 16.8)	1.1 (9.8 - 1.2)
UC, DUR	0.88 (0.88 – 0.88)	0.62 (0.62 – 0.62)	0.54 (0.54 – 0.54)
CAUTI rate	7.9 (7.5 - 8.4)	5.3 (5.2 – 5.8)	1.2 (1.1 - 1.3)
**Paediatric ICU**			
CL, DUR	0.40 (0.40 – 0.41)	0.50 (0.50 – 0.50)	0.47 (0.46 – 0.47)
CLABSI rate	9.5 (7.9 – 11.3)	6.1 (5.7 – 6.5)	1.8 (1.6 - 1.9)
MV, DUR	0.53 (0.53 – 0.54)	0.53 (0.53 – 0.53)	0.40 (0.40 – 0.40)
VAP rate	11.7 (10.2 - 13.5)	7.9 (7.4 – 8.4)	1.1 (9.0 - 1.2)
UC, DUR	0.34 (0.34 – 0.35)	0.31 (0.31 – 0.32)	0.23 (0.22 – 0.23)
CAUTI rate	6.6 (5.2 - 8.4)	5.6 (5.1 – 6.1)	3.1 (2.7 - 3.5)
**Neonatal ICU (weight 1501 to 2500 grams)**			
CL, DUR	0.09 (0.09 – 0.10)	0.21 (0.20 – 0.21)	0.18 (0.18 – 0.19)
CLABSI rate	7.8 (3.4 – 15.4)	4.8 (3.7 – 6.1)	0.7 (0.6 - 0.9)
MV, DUR	0.12 (0.11 – 0.13)	0.10 (0.10 – 0.11)	0.07 (0.07 – 0.07)
VAP rate	14.4 (8.7 - 22.5)	10.7 (8.4 – 13.4)	0.5 (0.2 - 0.9)

ICU, intensive care unit; CLABSI, central line-associated bloodstream infection; VAP, ventilator-associated pneumonia; CAUTI, catheter-associated urinary tract infection; DUR, device use ratio; INICC, International Nosocomial Infection Control Consortium; U.S. NSHN, National Healthcare Safety Network of the United States of America.

**Table 15:** **Benchmarking of antimicrobial resistance rates in this report against the report of the International Nosocomial Infection Control Consortium (2007–20012) and the report of the US National Healthcare Safety Network Data (2009–2010)**

	**This report resistance %**	**INICC 2007–2012 resistance %**	**NHSN 2009–2010 resistance, %**
**Pathogen, antimicrobial**	**(CLABSI)**	**(CLABSI)**	**(CLABSI)**
*Staphylococcus aureus*			
Oxacillin	92.7%	61.2%	54.6%
*Enterococcus faecalis*			
Vancomycin	5.0%	12.2%	9.5%
*Pseudomonas aeruginosa*			
Ciprofloxacine	35.3%	37.5%	30.5%
Piperacillin or piperacillin-tazobactam	27.6%	33.5%	17.4%
Amikacin	18.9%	42.8%	10.0%
Imipenem or meropenem	37.1%	42.4%	26.1%
*Klebsiella pneumoniae*			
Ceftriaxone or ceftazidime	55.7%	71.2%	28.8%
Imipenem or meropenem	6.3%	19.6%	12.8%
*Acinetobacter baumanii*			
Imipenem or meropenem	56.1%	66.3%	62.6%
*Escherichia Coli*			
Ceftriaxone or ceftazidime	55.2%	65.9%	19.0%
Imipenem or meropenem	4.4%	8.5%	1.9%
Ciprofloxacine	66.2%	69.3%	41.8%

CLABSI, central line-associated bloodstream infection.

## Discussion

Within the scientific literature addressing the burden of DA-HAIs in Turkey’s ICUs, in a recent study it was shown that the DA-HAI rates found in their setting were higher than the rates reported by the U.S. NHSN and INICC [11]. The CLABSI rate of our study was similar to the rate found in another study conducted in Turkey showing 11.8 CLABSIs per 1000 CL days [11]. Likewise, our CAUTI rate was similar to the findings of another study from ICUs in Turkey, showing 8.3 CAUTIs per 1000 UC days [12]. The VAP rate in our study was 21.4 per 1000 MV-days in adult and paediatric ICUs. Similarly, in 2008, Erdem et al. found a rate of 22.6 VAPs per 1000 MV-days [13], and Leblebicioglu et al. found a global VAP rate of 26.5 VAPs per 1000 MV-days in a multi-site study carried out in 12 hospitals in 2007 [12].

In our Turkish ICUs, DA-HAI rates and pooled DU ratios were higher than the Global INICC Report and U.S. NHSN’s data [5,6]. Likewise, the antimicrobial resistance rates found in our ICUs were higher than U.S. NHSN [4] and INICC [6] report rates for *Staphyloccocus aureus* as resistant to oxacillin, and for *Escherichia Coli* as resistant for imipenem. The resistance of *Escherichia Coli* to ciprofloxacin also higher than than U.S. NHSN [4], but similar to INICC report. [6] On the other hand, the resistance rates for *Pseudomonas aeruginosa* were higher in this study than U.S. NHSN report [4], but lower than the INICC reported resistance rates [6], as resistant to ciprofloxacin, piperacillin-tazobactam, amikacin and imipenem or meropenem; for *Escherichia Coli* as resistant to ceftriaxone and ceftazidime; and for *Klebsiella pneumonia* as resistant to ceftriaxone or ceftazidime. By contrast, the resistance rates for *Klebsiella pneumonia* and *Acinetobacter baumanii* as resistant to imipenem and meropenem, and *Enterococcus faecalis* as resistant to vancomycin, were lower in this study than in INICC and U.S. NHSN reports [4,6].

These high DA-HAI rates may reflect the typical ICU situation in hospitals in Turkey [14], and several reasons have been exposed to explain this fact [11,15]. Among the primary plausible causes, it can be mentioned that, in Turkey there are still no legally enforceable rules or regulations concerning the implementation of infection control programs, such as national infection control guidelines; yet, in the few cases in which there is a legal framework, adherence to the bundles is most irregular and hospital accreditation is not mandatory [16]. This situation is further emphasized by the fact that administrative and financial support is insufficient to fund infection control programmes, and invariably results in extremely low nurse-to-patient staffing ratios—which have proved to be highly connected to high DA-HAI rates in ICUs—, hospital over-crowding, lack of medical supplies, out-dated medical supplies and in an insufficient number of experienced nurses or trained healthcare workers [14].

In order to reduce the hospitalized patients’ risk of infection, DA-HAI surveillance is primary and essential, because it effectively describes and addresses the importance and characteristics of the threatening situation created by DA-HAIs. This must be followed by the implementation of practices aimed at DA-HAI prevention and control. Additionally, participation in INICC has played a fundamental role, not only in increasing the awareness of DA-HAI risks in the ICU, but also providing an exemplary basis for the institution of infection control practices. Finally, it is of utmost importance to restrict the administration of anti-infective in order to effectively control of antibiotic resistance.

The INICC programme is focused on surveillance of DA-HAIs in the ICU and surveillance of SSIs hospital wide; that is, healthcare settings (ICUs) and procedures (Surgical Procedures) with the highest healthcare-acquired rates, in which patients’ safety is most seriously threatened, due to their critical condition and exposure to invasive devices and surgical procedures [16]. Through the last 12 years, INICC has undertaken a global effort in America, Asia, Africa, Middle East, and Europe to respond to the burden of DA-HAIs, and has achieved extremely successful results, by increasing HH compliance, improving compliance with other infection control bundles and interventions as described in several INICC publications, and consequently reducing the rates of DA-HAI and mortality [6,17-21].

To compare a hospital's DA-HAI rates with the rates identified in this report, it is required that the hospital concerned start by collecting their data by applying the methods and methodology described for U.S. NHSN and INICC, and then calculate infection rates and DU ratios for the DA-HAI Module.

The particular and primary application of these data is to serve as a guide for the implementation of prevention strategies and other quality improvement efforts locally for the reduction of DA-HAI rates to the minimum possible level.

### Study limitations

The findings in this report are subject to at least two limitations. First, we did not consider the difference in time periods for the different data sources in the comparisons made with INICC and U.S. NHSN. Second, it is unfortunate that the study did not include data on possible changes in DA-HAIs in Turkey throughout the study period.

## Conclusions

In conclusion, the data presented in this report fortify the fact that DA-HAIs in Turkey pose a grave and many times concealed risk to patient safety, as compared to the developed world. It is INICC’s main goal to enhance infection control practices, by facilitating elemental, feasible and inexpensive tools and resources to tackle this problem effectively and systematically, leading to greater and stricter adherence to infection control programs and guidelines, and to the correlated reduction in DA-HAI and its adverse effects, in the hospitals participating in INICC, as well as at any other healthcare facility worldwide.

## References

[1] Laupland KB, Zygun DA, Doig CJ, Bagshaw SM, Svenson LW, Fick GH (2005). One-year mortality of bloodstream infection-associated sepsis and septic shock among patients presenting to a regional critical care system. Intensive Care Med.

[2] Fagon JY, Chastre J, Vuagnat A, Trouillet JL, Novara A, Gibert C (1996). Nosocomial pneumonia and mortality among patients in intensive care units. JAMA.

[3] Hughes JM (1988). Study on the efficacy of nosocomial infection control (SENIC Project): results and implications for the future. Chemotherapy.

[4] Sievert DM, Ricks P, Edwards JR, Schneider A, Patel J, Srinivasan A, Kallen A, Limbago B, Fridkin S (2013). Antimicrobial-resistant pathogens associated with healthcare-associated infections: summary of data reported to the National Healthcare Safety Network at the Centers for Disease Control and Prevention, 2009–2010. Infect Control Hosp Epidemiol.

[5] Dudeck MA, Horan TC, Peterson KD, Allen-Bridson K, Morrell G, Anttila A, Pollock DA, Edwards JR (2013). National Healthcare Safety Network report, data summary for 2011, device-associated module. Am J Infect Control.

[6] Rosenthal VD, Bijie H, Maki DG, Mehta Y, Apisarnthanarak A, Medeiros EA, Leblebicioglu H, Fisher D, Alvarez-Moreno C, Khader IA, Del Rocio Gonzalez Martinez M, Cuellar LE, Navoa-Ng JA, Abouqal R, Guanche Garcell H, Mitrev Z, Pirez Garcia MC, Hamdi A, Duenas L, Cancel E, Gurskis V, Rasslan O, Ahmed A, Kanj SS, Ugalde OC, Mapp T, Raka L, Yuet Meng C, le TA T, Ghazal S (2012). International Nosocomial Infection Control Consortium (INICC) report, data summary of 36 countries, for 2004–2009. Am J Infect Control.

[7] Rosenthal VD, Richtmann R, Singh S, Apisarnthanarak A, Kubler A, Viet-Hung N, Ramirez-Wong FM, Portillo-Gallo JH, Toscani J, Gikas A, Duenas L, El-Kholy A, Ghazal S, Fisher D, Mitrev Z, Gamar-Elanbya MO, Kanj SS, Arreza-Galapia Y, Leblebicioglu H, Hlinkova S, Memon BA, Guanche-Garcell H, Gurskis V, Alvarez-Moreno C, Barkat A, Mejia N, Rojas-Bonilla M, Ristic G, Raka L, Yuet-Meng C (2013). Surgical Site Infections, International Nosocomial Infection Control Consortium (INICC) Report, Data Summary of 30 Countries, 2005–2010. Infect Control Hosp Epidemiol.

[8] Rosenthal VD, Maki DG, Graves N (2008). The International Nosocomial Infection Control Consortium (INICC): goals and objectives, description of surveillance methods, and operational activities. Am J Infect Control.

[9] Horan TC, Andrus M, Dudeck MA (2008). CDC/NHSN surveillance definition of health care-associated infection and criteria for specific types of infections in the acute care setting. Am J Infect Control.

[10] Rosenthal VD, Maki DG, Mehta Y, Leblebicioglu H, Memish ZA, Al-Mousa HH, Balkhy H, Hu B, Alvarez-Moreno C, Medeiros EA, Apisarnthanarak A, Raka L, Cuellar LE, Ahmed A, Navoa-Ng JA, El-Kholy AA, Kanj SS, Bat-Erdene I, Duszynska W, Van Truong N, Pazmino LN, See-Lum LC, Fernandez-Hidalgo R, Di-Silvestre G, Zand F, Hlinkova S, Belskiy V, Al-Rahma H, Luque-Torres MT, Bayraktar N (2014). International Nosocomial Infection Control Consortium (INICC) report, data summary of 43 countries for 2007-2012. Device-associated module. Am J Infect Control.

[11] Dogru A, Sargin F, Celik M, Sagiroglu AE, Goksel MM, Sayhan H (2010). The rate of device-associated nosocomial infections in a medical surgical intensive care unit of a training and research hospital in Turkey: one-year outcomes. Jpn J Infect Dis.

[12] Leblebicioglu H, Rosenthal VD, Arikan OA, Ozgultekin A, Yalcin AN, Koksal I, Usluer G, Sardan YC, Ulusoy S (2007). Device-associated hospital-acquired infection rates in Turkish intensive care units. Findings of the International Nosocomial Infection Control Consortium (INICC). J Hosp Infect.

[13] Erdem I, Ozgultekin A, Inan AS, Dincer E, Turan G, Ceran N, Ozturk Engin D, Senbayrak Akcay S, Akgun N, Goktas P (2008). Incidence, etiology, and antibiotic resistance patterns of gram-negative microorganisms isolated from patients with ventilator-associated pneumonia in a medical-surgical intensive care unit of a teaching hospital in istanbul, Turkey (2004–2006). Jpn J Infect Dis.

[14] Inan A, Ozgultekin A, Akcay SS, Engin DO, Turan G, Ceran N, Dincer E, Aksaray S, Goktas P, Erdem I (2012). Alterations in bacterial spectrum and increasing resistance rates in isolated microorganisms from device-associated infections in an intensive care unit of a teaching hospital in Istanbul (2004–2010). Jpn J Infect Dis.

[15] Hugonnet S, Harbarth S, Sax H, Duncan RA, Pittet D (2004). Nursing resources: a major determinant of nosocomial infection?. Curr Opin Infect Dis.

[16] Rosenthal VD, Maki DG, Salomao R, Moreno CA, Mehta Y, Higuera F, Cuellar LE, Arikan OA, Abouqal R, Leblebicioglu H (2006). Device-associated nosocomial infections in 55 intensive care units of 8 developing countries. Ann Intern Med.

[17] Rosenthal VD, Maki DG, Rodrigues C, Alvarez-Moreno C, Leblebicioglu H, Sobreyra-Oropeza M, Berba R, Madani N, Medeiros EA, Cuellar LE, Mitrev Z, Duenas L, Guanche-Garcell H, Mapp T, Kanj SS, Fernandez-Hidalgo R (2010). Impact of International Nosocomial Infection Control Consortium (INICC) strategy on central line-associated bloodstream infection rates in the intensive care units of 15 developing countries. Infect Control Hosp Epidemiol.

[18] Rosenthal VD, Ramachandran B, Villamil-Gomez W, Armas-Ruiz A, Navoa-Ng JA, Matta-Cortes L, Pawar M, Nevzat-Yalcin A, Rodriguez-Ferrer M, Yildizdas RD, Menco A, Campuzano R, Villanueva VD, Rendon-Campo LF, Gupta A, Turhan O, Barahona-Guzman N, Horoz OO, Arrieta P, Brito JM, Tolentino MC, Astudillo Y, Saini N, Gunay N, Sarmiento-Villa G, Gumus E, Lagares-Guzman A, Dursun O (2012). Impact of a multidimensional infection control strategy on central line-associated bloodstream infection rates in pediatric intensive care units of five developing countries: findings of the International Nosocomial Infection Control Consortium (INICC). Infection.

[19] Rosenthal VD, Alvarez-Moreno C, Villamil-Gomez W, Singh S, Ramachandran B, Navoa-Ng JA, Duenas L, Yalcin AN, Ersoz G, Menco A, Arrieta P, Bran-de Casares AC, de Jesus Machuca L, Radhakrishnan K, Villanueva VD, Tolentino MC, Turhan O, Keskin S, Gumus E, Dursun O, Kaya A, Kuyucu N (2012). Effectiveness of a multidimensional approach to reduce ventilator-associated pneumonia in pediatric intensive care units of 5 developing countries: International Nosocomial Infection Control Consortium findings. Am J Infect Control.

[20] Rosenthal VD, Rodriguez-Calderon ME, Rodriguez-Ferrer M, Singhal T, Pawar M, Sobreyra-Oropeza M, Barkat A, Atencio-Espinoza T, Berba R, Navoa-Ng JA, Duenas L, Ben-Jaballah N, Ozdemir D, Ersoz G, Aygun C (2012). Findings of the International Nosocomial Infection Control Consortium (INICC), Part II: Impact of a multidimensional strategy to reduce ventilator-associated pneumonia in neonatal intensive care units in 10 developing countries. Infect Control Hosp Epidemiol.

[21] Rosenthal VD, Rodrigues C, Alvarez-Moreno C, Madani N, Mitrev Z, Ye G, Salomao R, Ulger F, Guanche-Garcell H, Kanj SS, Cuellar LE, Higuera F, Mapp T, Fernandez-Hidalgo R (2012). Effectiveness of a multidimensional approach for prevention of ventilator-associated pneumonia in adult intensive care units from 14 developing countries of four continents: findings of the International Nosocomial Infection Control Consortium. Crit Care Med.

